# Association of Prenatal Exposure to Valproate and Other Antiepileptic Drugs With Intellectual Disability and Delayed Childhood Milestones

**DOI:** 10.1001/jamanetworkopen.2020.25570

**Published:** 2020-11-10

**Authors:** Christine Aarenstrup Daugaard, Lars Pedersen, Yuelian Sun, Julie Werenberg Dreier, Jakob Christensen

**Affiliations:** 1 Department of Neurology, Aarhus University Hospital, Aarhus, Denmark; 2 Department of Clinical Epidemiology, Aarhus University Hospital, Aarhus, Denmark; 3 The National Centre for Register-Based Research, Department of Economics and Business Economics, Business and Social Sciences, Aarhus University, Aarhus, Denmark; 4 The Centre for Integrated Register-Based Research, Aarhus University, Aarhus, Denmark

## Abstract

**Importance:**

There is concern about neurodevelopmental outcomes associated with prenatal exposure to valproate and other antiepileptic drugs (AEDs) among children of mothers with or without epilepsy.

**Objective:**

To study the risk of intellectual disability and delayed development in childhood milestones among children of women who used valproate or other AEDs during pregnancy.

**Design, Setting, and Participants:**

This population-based cohort study analyzed information on use of AEDs from the Danish National Prescription Registry and register diagnoses from the Danish Psychiatric Central Research Register and Danish National Patient Registry. The study included all live-born singletons in Denmark from January 1, 1997, to December 31, 2011. Data were analyzed in April 2020.

**Exposures:**

Prenatal exposure to maternal valproate and other AEDs.

**Main Outcomes and Measures:**

The main measures were adjusted Cox regression estimates of hazard ratios (aHRs) for intellectual disability and a combined outcome of intellectual disability with delayed childhood milestones.

**Results:**

A total of 913 302 children (468 708 [51.3%] boys; mean [SD] age, 10.3 [4.4] years and median [interquartile range] age, 10.1 [6.5-14.0] years at final follow-up) were identified and contributed more than 10.2 million person-years of observation, including 580 children exposed to valproate (302 [51.3%] boys). At end of follow-up, 6958 children (0.8%) were identified as having intellectual disability and 14 967 children (1.6%) were identified as having intellectual disability with delayed childhood milestones. Compared with offspring not exposed to valproate prenatally, offspring of women who used valproate during pregnancy had increased risk of intellectual disability (aHR, 4.48; 95% CI, 2.97-6.76) and intellectual disability with delayed childhood milestones (aHR, 6.07; 95% CI, 4.67-7.89). Among mothers with epilepsy, offspring exposed prenatally to valproate had increased risk of intellectual disability (aHR, 1.95; 95% CI, 1.21-3.14) and intellectual disability with delayed childhood milestones (aHR, 3.07; 95% CI, 2.24-4.20) compared with offspring without prenatal exposure. Compared with offspring without prenatal exposure to AEDs, increased risk of intellectual disability was identified in children with prenatal exposure to maternal monotherapy use of carbamazepine (aHR, 3.84; 95% CI, 2.32-6.38), clonazepam (aHR, 2.41; 95% CI, 1.09-5.35), and oxcarbazepine (aHR, 3.70; 95% CI, 2.11-6.51) but not lamotrigine (aHR, 1.33; 95% CI, 0.71-2.48).

**Conclusions and Relevance:**

These findings suggest that prenatal exposure to valproate was associated with increased risk of intellectual disability and delayed childhood milestones. Statistically significant associations were also found for prenatal exposure to other AEDs. These findings suggest that women of childbearing potential may need to be counseled on use of AEDs.

## Key Points

**Question:**

Is prenatal exposure to valproate or other antiepileptic drugs associated with increased risk of intellectual disability and delayed development in childhood milestones?

**Findings:**

In this cohort study of 913 302 Danish children, prenatal valproate exposure was associated with 4.5-fold increased risk of intellectual disability and 6-fold increased risk of combined outcome of intellectual disability and delayed childhood milestones compared with children with no prenatal valproate exposure. Other antiepileptic drugs, including carbamazepine, clonazepam, and oxcarbazepine, were also found to be associated with increased risk of intellectual disability.

**Meaning:**

These findings add to the evidence for risk in the use of valproate among pregnant women and all women of childbearing potential and suggest risks in the use of other antiepileptic drugs by these populations.

## Teaser

This cohort study evaluates risk of intellectual disability and delayed childhood milestones among children exposed prenatally to valproate and other antiepileptic drugs.

## Introduction

Intellectual disability is a severe neurodevelopmental disorder, characterized by inhibited mental development and impairment of cognitive abilities with an IQ usually less than 70.^1,2^ The use of antiepileptic drugs (AEDs), including valproate, by pregnant women has been associated with adverse neurodevelopment in their children.^3^ Studies have also found that among women with epilepsy who used valproate while pregnant, offspring were more likely to have IQs less than 70 compared with offspring of women with epilepsy who used other AEDs while pregnant.^4,5,6^ In addition, in utero exposure to valproate has been associated with increased risk of impaired academic performance,^7^ congenital malformations,^8^ attention-deficit/hyperactivity disorder,^9^ and autism.^10^

A 2019 study^4^ assessed neurodevelopment in offspring of women who had epilepsy and used valproate while pregnant, comparing the associated risk of adverse neurodevelopment with that among offspring of women with epilepsy who used other AEDs while pregnant. However, no studies to our knowledge have compared the risk of intellectual disability among children exposed to AEDs before birth with that of children not exposed to AEDs before birth, and we therefore assessed this risk in a large, nationwide study in Denmark. To give a fuller description of adverse events that may be associated with prenatal exposure to AEDs, we included delayed development in childhood milestones in our risk assessment.

## Methods

All data in this cohort study were analyzed at Statistics Denmark using encrypted identification numbers with no contact with the individuals. By Danish law, analysis of anonymous data as part of health science registry research projects does not require ethical review board approval or informed consent. The study was approved by the Danish Data Protection Agency. The study follows the Strengthening the Reporting of Observational Studies in Epidemiology (STROBE) reporting guideline.

### Population and Exposure

We conducted a population-based cohort study and included in the study population all singleton children born alive in Denmark between January 1, 1997, and December 31, 2011. In Denmark, every individual is assigned a unique personal identification number in the Danish Civil Registration System when born in or after immigrating to Denmark. This number is used to ensure complete individual linkage among registers.^11^

The Danish Prescription Register^12^ contains unique information on all redeemed prescriptions purchased since January 1, 1996. We defined the exposure window as the period from 30 days before a child’s estimated day of conception to the day of birth and included children with an estimated time of conception after February 1, 1996. Exposure to AEDs was defined as any redeemed prescription for the mother within the exposure window with the Anatomical Therapeutic Chemical code N03A (for antiepileptic drugs), including N03AG01 (for valproate) and N05BA09 (for clobazam). Exposure during this window was considered prenatal exposure for the child.

Monotherapy was defined as a mother redeeming prescriptions for only 1 type of AED within the exposure window, and polytherapy was defined as a mother redeeming prescriptions for more than 1 type of AED within the exposure window. We also assessed risk of intellectual disability and of combined outcome of intellectual disability with delayed childhood milestones among children exposed to frequently used AEDs (ie, carbamazepine, clonazepam, lamotrigine, and oxcarbazepine).^13^ Mean daily dose of AED was estimated from the total amount of AED redeemed from 30 days before pregnancy until birth, divided by the number of days in the same period.

### Information on Intellectual Disability and Covariates

The Danish Psychiatric Central Research Register^14^ and Danish National Patient Registry^15^ were used to identify children who were diagnosed, using *International Statistical Classification of Diseases and Related Health Problems, Tenth Revision (ICD-10)*,^1^ with intellectual disability (*ICD-10* codes F70-F79) or delayed development in childhood milestones (*ICD-10* code R62.0). Information on parity was obtained from the Danish Medical Birth Registry.^16^ We used the Danish National Patient Registry^15^ to identify children with congenital malformations (*ICD-10* codes Q0-Q99)^15^ and mothers diagnosed with epilepsy before the birth of the child (*International Classification of Diseases, Eighth Revision [ICD-8]*^17^ code 345 and *ICD-10* codes G40 and G41). We identified pregnant women diagnosed with psychiatric disorders before the birth of their children (*ICD-8* codes 290-315 and *ICD-10* codes F0.00-F99.9) from the Danish Psychiatric Central Research Register.^14^ In addition, we assessed which underlying disorders were being treated with valproate in pregnancies in which the mother did not have an epilepsy diagnosis but was taking the medication (eAppendix in the Supplement).

### Statistical Analysis

Children were followed from birth to first diagnosis of intellectual disability or delayed childhood milestone, death, emigration, or December 31, 2015, whichever came first. We used Cox regression to estimate hazard ratios (HRs), including 95% CIs, for intellectual disability and combined outcome of intellectual disability with delayed childhood milestones for children with prenatal valproate exposure. Age of the child was used as the underlying time scale, and separate baseline diagnostic rates (stratum) were used for each birth-year group to adjust for the decreasing use of valproate in pregnancy and the increasing prevalence of diagnoses of intellectual disability and delayed childhood milestones. The proportional hazards assumption was evaluated for all variables by comparing estimated log-minus-log survivor curves over the different categories of variables investigated. Evaluation of the proportional hazards assumption was performed by visual inspection, and no violation was detected. Control for the lack of independence of children within the same family was obtained using a robust Huber-White variance estimator. For intellectual disability and delayed childhood milestones, HRs were adjusted (aHRs) for risk factors, including maternal age at conception (ie, ages 15-24, 25-29, 30-34, and ≥35 years), maternal psychiatric diagnosis status (ie, yes or no), maternal epilepsy status (ie, yes or no), maternal diabetes status (ie, yes or no), sex of the child, year of birth of child, and parity (ie, 1, 2, or ≥3). We calculated the cumulative incidence of intellectual disability and the combined outcome of intellectual disability with delayed childhood milestones, with death as a competing event.

To assess possible confounding by indication, we compared risk of intellectual disability and of combined outcome in offspring of women who used valproate while pregnant with that of women who discontinued use of valproate at least 30 days before the estimated day of conception (ie, previous users). We also estimated outcomes (1) in offspring of women exposed to valproate during pregnancy compared with women exposed to lamotrigine during pregnancy, (2) after stratifying risk by monotherapy vs polytherapy use of AEDs, (3) after stratifying by AED dose in monotherapy, and (4) after excluding children with congenital malformations.

Data analyses were performed using Stata statistical software version 12 (StataCorp). A 2-sided χ^2^ test was used to evaluate *P* values, and the level of statistical significance was .05. Data were analyzed in April 2020.

## Results

We identified 913 302 children (468 708 [51.3%] boys; mean [SD] age 10.3 [4.4] years and median [interquartile range] age 10.1 [6.5-14.0] years at final follow-up), who contributed more than 10.2 million person-years of observation; 28 499 children (3.1%) were lost to follow-up, of whom 4471 children (0.5%) were lost due to death and 24 028 children (2.6%) were lost due to emigration.

Of 913 302 children in this cohort, 580 children (302 [51.3%] boys) were exposed to valproate before birth (monotherapy and polytherapy combined) (Table 1). Compared with mothers who did not use valproate during pregnancy, mothers who used valproate during pregnancy were younger and more often diagnosed with epilepsy and psychiatric disorders. Compared with children who were not exposed before birth, children exposed to valproate before birth were more often diagnosed with congenital malformations during the first year of life (Table 1).

**Table 1.** Characteristics of Study Population

Characteristic	No. (%)	
	Children with prenatal valproate exposure (n = 580)	Children without prenatal valproate exposure (n = 912 722)
**Maternal**		
Age, y		
15-24	105 (18.1)	122 304 (13.4)
25-29	189 (32.6)	310 282 (34.0)
30-34	196 (33.8)	324 315 (35.5)
≥35	90 (15.5)	155 821 (17.1)
Epilepsy diagnosis status		
No	64 (11.0)	905 618 (99.2)
Yes	516 (89.0)	7104 (0.8)
Psychiatric diagnosis status		
No	506 (87.2)	841 309 (92.2)
Yes	74 (12.8)	71 413 (7.8)
Diabetes diagnosis status		
No	564 (97.2)	889 727 (97.5)
Yes	16 (2.8)	22 995 (2.5)
Parity		
1	241 (47.3)	392 798 (43.0)
2	174 (34.1)	334 259 (36.6)
≥3	87 (17.1)	172 510 (18.9)
Missing	8 (1.6)	13 155 (1.4)
**Child**		
Sex		
Girls	278 (48.7)	444 316 (48.7)
Boys	302 (51.3)	468 406 (51.3)
Birth year		
1997-1999	189 (32.6)	183 871 (20.1)
2000-2002	126 (21.7)	186 165 (20.4)
2003-2005	116 (20.0)	183 097 (20.1)
2006-2008	88 (15.2)	184 049 (20.2)
2009-2011	61 (10.5)	175 540 (19.2)
Malformations in first year of life		
No	512 (88.3)	873 710 (95.7)
Yes	68 (11.7)	39 012 (4.3)

A total of 6958 children (0.8%) were identified with intellectual disability, and 14 967 children (1.6%) were identified with combined outcome of intellectual disability with delayed childhood milestones (Table 2). Among 912 722 children not exposed to valproate before birth, we identified 6935 children (0.8%) diagnosed with intellectual disability and 14 909 children (1.6%) diagnosed with combined outcome. Of 580 children who were exposed to valproate before birth, 23 children (4.0%) were diagnosed with intellectual disability and 58 children (10.0%) had the combined diagnosis.

**Table 2.** Risk of Intellectual Disability and Combined Outcome of Intellectual Disability and Delayed Childhood Milestones Associated With Prenatal Valproate Exposure

Exposure	Live births, No.	Person-years at risk, No.	Diagnoses, No.	Incidence, No. (95% CI), cases per 1000 person-years	HR (95% CI)	
					Crude	Adjusted^a^
**Intellectual disability**						
Exposed to valproate	580	7153	23	3.2 (2.1-4.8)	4.69 (3.11-7.07)	4.48 (2.97-6.76)
Not exposed to valproate	912 722	10 304 108	6935	0.7 (0.7-0.7)	1 [Reference]	1 [Reference]
**Combined outcome**						
Exposed to valproate	580	6816	58	8.5 (6.6-7.9)	6.08 (4.68-7.88)	6.07 (4.67-7.89)
Not exposed to valproate	912 722	10 234 967	14 909	1.5 (1.4-1.5)	1 [Reference]	1 [Reference]

Abbreviation: HR, hazard ratio.

^a^ Adjusted for maternal age at conception, maternal psychiatric history, maternal epilepsy status, maternal diabetes status, sex of child, year of birth of child, and parity.

Children exposed to valproate prenatally had increased risk of intellectual disability (aHR, 4.48; 95% CI, 2.97-6.76) and combined outcome (aHR, 6.07; 95% CI, 4.67-7.89) compared with children who were not exposed prenatally (Table 2). Of 7620 children born to women with epilepsy, 516 children (6.8%) were exposed to valproate before birth, which was associated with a nearly 2-fold increase in risk of intellectual disability (aHR, 1.95; 95% CI, 1.21-3.14) and more than 3-fold increase in risk of combined outcome (aHR, 3.07; 95% CI, 2.24-4.20) compared with 7104 children born to mothers with epilepsy who did not use valproate during pregnancy (Table 3). In point estimates among 64 children born to women without epilepsy and exposed to valproate before birth, there was no statistically significant difference in risk of intellectual disability (aHR, 1.81; 95% CI, 0.25-13.0) or combined outcome (aHR, 1.92; 95% CI, 0.48-7.76) compared with 905 618 children born to mothers without epilepsy who did not use valproate during pregnancy.

**Table 3.** Risk of Intellectual Disability and Combined Outcome of Intellectual Disability and Delayed Childhood Milestones, Stratified by Epilepsy Diagnosis of Mother

Maternal epilepsy status	Live births, No.	Incidence, No. (95% CI), cases per 1000 person-years	HR (95% CI)	
			Crude	Adjusted^a^
**Intellectual disability**				
Epilepsy				
Prenatal valproate exposure	516	3.5 (2.3-5.3)	1.99 (1.25-3.16)	1.95 (1.21-3.14)
No prenatal valproate exposure	7104	1.7 (1.4-2.0)	1 [Reference]	1 [Reference]
No epilepsy				
Prenatal valproate exposure^b^	64	1.2 (0.2-8.4)	1.74 (0.25-12.2)	1.81 (0.25-13.0)
No prenatal valproate exposure	905 618	0.7 (0.7-0.7)	1 [Reference]	1 [Reference]
**Combined outcome**				
Epilepsy				
Prenatal valproate exposure	516	9.4 (7.2-12.2)	3.03 (2.25-4.07)	3.07 (2.24-4.20)
No prenatal valproate exposure	7104	3.4 (3.0-3.9)	1 [Reference]	1 [Reference]
No epilepsy				
Prenatal valproate exposure^b^	64	2.4 (0.6-9.6)	1.84 (0.47-7.24)	1.92 (0.48-7.76)
No prenatal valproate exposure	905 618	1.4 (1.4-1.5)	1 [Reference]	1 [Reference]

Abbreviation: HR, hazard ratio.

^a^ Adjusted for maternal age at conception, maternal psychiatric history, maternal diabetes status, sex of child, year of birth of child, and parity.

^b^ Among these 64 pregnancies, there were too few incidences of other diagnoses of the mother to allow further analyses of other indications for valproate treatment.

Cumulative risk increased up to age 18 years for intellectual disability (Figure, part A) and combined outcome (Figure, part B). At age 18 years, cumulative incidence of intellectual disability was 1.35% (95% CI, 1.29%-1.42%) in children not exposed to valproate prenatally, 5.99% (95% CI, 3.43%-9.52%) in children exposed to valproate prenatally, and 3.15% (95% CI, 2.50%-3.90%) in children of mothers with epilepsy who did not use valproate during pregnancy. At age 18 years, cumulative incidence of combined outcome was 2.20% (95% CI, 2.13%-2.26%) in children not exposed to valproate prenatally, 12.03% (95% CI, 8.81%-15.79%) in children exposed to valproate prenatally, and 4.63% (95% CI, 3.95%-5.40%) in children of mothers with epilepsy who did not use valproate during pregnancy.

**Figure.**
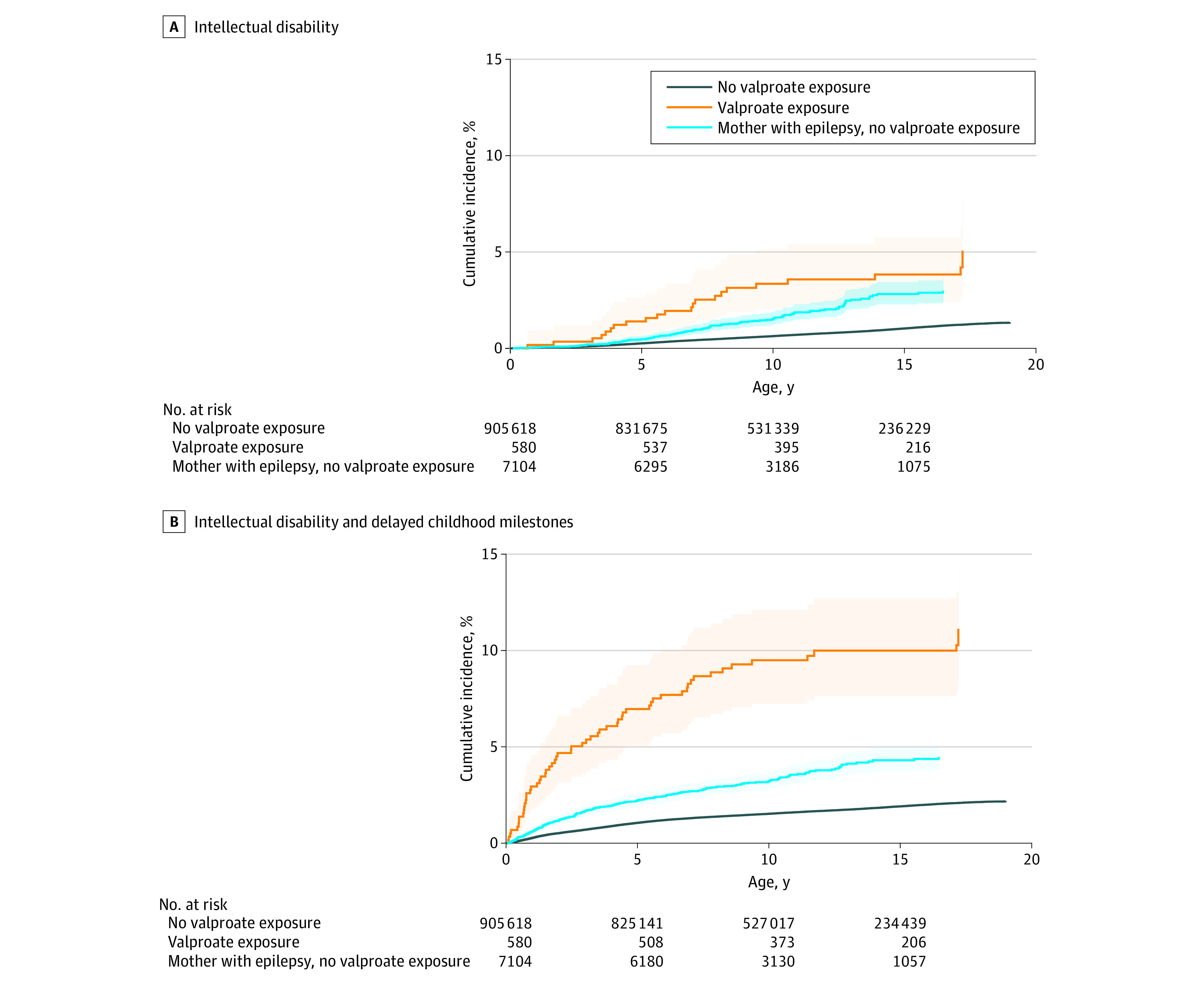
Cumulative Incidence in Children With or Without Prenatal Exposure to Valproate A, Cumulative incidence of intellectual disability, with death as a competing event. Cohorts are not mutually exclusive. Shaded areas indicate 95% CIs. B, Cumulative incidence of combined outcome of intellectual disability with delayed childhood milestones, with death as a competing event. Cohorts are not mutually exclusive. Shaded areas indicate 95% CIs.

Among 431 children of women who used valproate in monotherapy during pregnancy, risk of intellectual disability was increased more than 4-fold (aHR, 4.42; 95% CI, 2.75-7.11) and risk of combined outcome was increased more than 5-fold (aHR, 5.28; 95% CI, 3.82-7.28) compared with offspring of women who did not use AEDs during pregnancy (Table 4). Compared with children not exposed to AEDs before birth, risk of intellectual disability and combined outcome associated with exposure to monotherapy with other AEDs was increased. For carbamazepine, risk was increased nearly 4-fold for intellectual disability (aHR, 3.84; 95% CI, 2.32-6.38) and approximately 2.5-fold for combined outcome (aHR, 2.49; 95% CI, 1.58-3.94); for clonazepam, risk was increased nearly 4-fold for intellectual disability (aHR, 3.84; 95% CI, 2.32-6.38) and approximately 3-fold for combined outcome (aHR, 2.99; 95% CI, 1.80-4.96); and for oxcarbazepine, risk was increased more than 3.5 fold for intellectual disability (aHR, 3.70; 95% CI, 2.11-6.51) and approximately 2.5-fold for combined outcome (aHR, 2.51; 95% CI, 1.54-4.10). There was no increased risk of intellectual disability in children whose mothers used lamotrigine as monotherapy (aHR, 1.33; 95% CI, 0.71-2.48), but risk of combined outcome was increased nearly 1.5-fold (aHR, 1.45; 95% CI, 1.01-2.09) compared with offspring of women who did not use AEDs during pregnancy (Table 4).

**Table 4.** Risk of Intellectual Disability and Combined Outcome of Intellectual Disability and Delayed Childhood Milestones Associated With Prenatal AED Exposure

Monotherapy AED exposure	Live births, No.	Person-years at risk, No.	Diagnoses, No.	Incidence, No. (95% CI), cases per 1000 person-years	HR (95% CI)	
					Crude	Adjusted^a^
**Intellectual disability**						
Valproate	431	5398	17	3.1 (2.0-5.1)	4.65 (2.89-7.49)	4.42 (2.75-7.11)
Carbamazepine	423	5650	15	2.7 (1.6-4.4)	3.88 (2.34-6.44)	3.84 (2.32-6.38)
Clonazepam	314	3744	6	1.6 (0.7-3.6)	2.41 (1.09-5.34)	2.41 (1.09-5.35)
Oxcarbazepine	372	4656	12	2.6 (1.6-4.5)	3.82 (2.17-6.72)	3.70 (2.11-6.51)
Lamotrigine	1383	12 244	10	0.8 (0.4-1.5)	1.30 (0.70-2.42	1.33 (0.71-2.48)
No AED	899 941	10 176 941	6744	0.7 (0.6-0.7)	1 [Reference]	1 [Reference]
**Combined outcome**						
Valproate	431	5184	38	7.3 (5.3-10.1)	5.35 (3.89-7.38)	5.28 (3.82-7.28)
Carbamazepine	423	5582	18	3.2 (2.0-5.1)	2.42 (1.53-3.83)	2.49 (1.58-3.94)
Clonazepam	314	3648	15	4.1 (2.5-6.8)	2.92 (1.76-4.84)	2.99 (1.80-4.96)
Oxcarbazepine	372	4614	16	3.5 (2.1-5.7)	2.55 (1.57-4.13)	2.51 (1.54-4.10)
Lamotrigine	1383	12 104	29	2.4 (1.7-3.4)	1.50 (1.04-2.16)	1.45 (1.01-2.09)
No AED	899 941	10 109 575	14 502	1.4 (1.4-1.5)	1 [Reference]	1 [Reference]

Abbreviations: AED, antiepileptic drug; HR, hazard ratio.

^a^ Adjusted for maternal age at conception, maternal psychiatric history, maternal diabetes status, sex of child, year of birth of child, and parity.

### Sensitivity Analyses

Compared with prenatal exposure to lamotrigine, risk of intellectual disability associated with prenatal exposure in monotherapy was increased approximately 5-fold for valproate (aHR, 4.91; 95% CI, 2.09-11.55), more than 3-fold for carbamazepine (aHR, 3.30; 95% CI, 1.51-7.24), and approximately 3.5-fold for oxcarbazepine (aHR, 3.53; 95% CI, 1.40-8.90). Compared with children exposed to lamotrigine before birth, risk of intellectual disability associated with prenatal exposure to clonazepam was not significantly increased (aHR, 1.90; 95% CI, 0.70-5.17) (eTable 1 in the Supplement). Similar findings were identified for combined outcome for all AEDs (eTable 1 in the Supplement). Compared with 719 children of women who used valproate before but not during pregnancy, there was no increased risk of intellectual disability in 580 offspring of women who used valproate during pregnancy (aHR, 1.94; 95% CI, 0.99-3.80), but there was increased risk of combined outcome (aHR, 2.25; 95% CI, 1.46-3.47) (eTable 2 in the Supplement).

Increase in risk of intellectual disability, compared with 899 941 children of women who did not use an AED during pregnancy, was similar for 431 children of women who used valproate as monotherapy during pregnancy and 149 children of women who used valproate as polytherapy during pregnancy (aHR, 4.42; 95% CI, 2.75-7.11 vs aHR, 4.99; 95% CI, 2.21-11.30) (eTable 3 in the Supplement). For combined outcome, risk estimates were not different for polytherapy vs monotherapy (aHR, 8.95; 95% CI, 5.71-14.04 vs aHR, 5.28; 95% CI, 3.82-7.28; *P* for comparison = .06) (eTable 3 in the Supplement).

Estimates suggested a dose-response association; compared with 899 941 children of women who were not exposed to AEDs during pregnancy, there was an increase in risk of intellectual disability among 213 children of women who used valproate in high-dose monotherapy during pregnancy (>750 mg/d) (aHR, 7.96; 95% CI, 4.80-13.19), but there was no increase in risk among 218 children of women who used valproate in low-dose monotherapy (≤750 mg/d) during pregnancy (aHR, 1.01; 95% CI, 0.25-4.08; *P* for comparison = .006) (eTable 4 in the Supplement). Similar dose-response associations were identified for combined outcome, with an increased risk for high-dose use (aHR, 9.47; 95% CI: 6.64-13.49) and no increased risk for low-dose use (aHR, 1.57; 95% CI, 0.70-3.50; *P* for comparison < .001) (eTable 4 in the Supplement). When excluding 39 080 children with congenital malformations, risk among 512 children exposed to valproate before birth remained significantly increased for intellectual disability (aHR, 4.06; 95% CI, 2.48-6.63) and combined outcome (aHR 4.85; 95% CI, 3.49-6.74) compared with 873 710 children not exposed to valproate before birth (eTable 5 in the Supplement).

## Discussion

This cohort study identified an association between prenatal exposure to valproate and risk of intellectual disability and of combined outcome of intellectual disability with delayed childhood milestones. The association between prenatal exposure to valproate and intellectual disability was also found compared with children exposed prenatally to lamotrigine. Furthermore, the association persisted after adjusting for a range of potential confounders (ie, maternal psychiatric disorders, maternal epilepsy and diabetes status, maternal age, sex of child, year of birth of child, and parity) and showed an association with high-dose exposure but not with low-dose exposure.

Analyses of other types of AEDs suggested that they were also associated with an increased risk of intellectual disability and delayed childhood milestones in children of women who used these drugs while pregnant. We found increased risk associated with carbamazepine, clonazepam, and oxcarbazepine, associations that have not been previously described, to our knowledge. For lamotrigine used in monotherapy, no association was found with risk of intellectual disability but an association was found for combined outcome. Further research could include exposure to AEDs not assessed in this study, such as levetiracetam and topiramate.^18^

Prenatal exposure to higher valproate dosages was associated with increased risk of intellectual disability while exposure to lower doses was not. These results agree with a 2018 study^19^ and a 2013 study^20^ suggesting that teratogenic and neurodevelopmental events were mainly associated with high daily doses of valproate, although our cutoff point between low dose and high dose (750 mg/d) was lower than that in in the 2013 study^20^ (1000 mg/d).

In agreement with previous studies,^21,22,23^ we found that prenatal valproate exposure was associated with increased incidence of congenital malformations in the first year of life. However, increased risk of intellectual disability and combined outcome remained significant after excluding all children diagnosed with congenital malformations, suggesting that the risk was not confined to children with valproate-induced congenital malformations.

### Strengths and Limitations

Strengths of this study include its population-based design and rate of follow-up, with nearly 97% of the identified cohort followed. The study combined data on diagnoses of intellectual disability and delayed childhood milestones from hospital-based registers, allowing more general assessment of potential consequences for neurodevelopment compared with uncombined diagnoses, especially in early life. For example, the combination of outcomes may allow identification of milder symptoms of cognitive impairment not captured by intellectual disability diagnosis alone.^24^

This observational cohort study also has several limitations. First, valproate is relatively contraindicated in women of fertile age, and it is likely that women who maintain valproate prescription during pregnancy differ from women who do not use valproate. We cannot exclude the possibility that the association found between maternal valproate use in pregnancy and intellectual disability and delayed childhood milestone risk in offspring may be, at least in part, due to residual confounding from maternal diseases, because the registries may not capture all women with epilepsy and psychiatric disorders. Although a 2011 study^14^ validated psychiatric diagnoses (eg, schizophrenia, single-episode depression, dementia, and autism) in the Danish Psychiatric Central Research Register, no study to our knowledge has validated the clinical diagnosis of intellectual disability against research diagnoses and no study of completeness of intellectual disability diagnoses exists. However, a validation study^25^ of epilepsy diagnosis in the Danish National Patient Register found relatively high validity and completeness of diagnosis, suggesting that this register captures most maternal epilepsy diagnoses. Second, data used in this study were based on register data,^26^ and we do not know whether the women used the medication registered to them or how much valproate was actually used. However, for AEDs, agreement between self-reports and dispensing data is high.^27,28,29^ In the analyses associated with AED dose, we estimated mean daily AED dose on the basis of the total amount of redeemed medication throughout pregnancy. It is unlikely that individuals would repeatedly purchase medication if it was not consumed, and it is therefore reasonable to assume that in the high-dose group, the proportion of women not consuming medication was low. Third, we did not control for other types of medications beyond AED that mothers may have redeemed during pregnancy, so part of the observed risk could be attributed to other medications. For example, information on use of folic acid, which may affect neurodevelopment,^30,31^ was not included in this study. However, including other drugs in the analyses may have yielded spurious associations due to the number, combinations, and variety of the drugs. Fourth, statistical precision of the analyses of dose was low due to the small number of exposed children, and the results of this analysis and sensitivity analyses in the study should therefore be interpreted with caution.

## Conclusions

This cohort study found that use of valproate, as well as several other AEDs, by mothers during pregnancy was associated with significantly increased risk of intellectual disability and delayed childhood milestones in children from those pregnancies, with clear implications for women of childbearing potential. These results warrant further studies using data from larger observational cohorts, with sufficient follow-up of offspring neurodevelopment given that associations cannot be studied in randomized clinical trials.

## Notes

Supplement. **eAppendix.** Underlying Disorders for Valproate Use During Pregnancy Without Maternal Epilepsy Diagnosis **eTable 1.** Risk of Intellectual Disability and Combined Outcome of Intellectual Disability and Delayed Childhood Milestones Associated With Prenatal Antiepileptic Drugs in Monotherapy Exposure vs Prenatal Lamotrigine in Monotherapy Exposure **eTable 2.** Risk of Intellectual Disability and Combined Outcome of Intellectual Disability and Delayed Childhood Milestones Associated With Valproate Use During Pregnancy vs Valproate Use Prior to Pregnancy **eTable 3.** Risk of Intellectual Disability and Combined Outcome of Intellectual Disability and Delayed Childhood Milestones Associated With Prenatal Antiepileptic Drug (AED) Exposure in Polytherapy and Monotherapy vs No Prenatal AED Exposure **eTable 4.** Risk of Intellectual Disability and Combined Outcome of Intellectual Disability and Delayed Childhood Milestones Associated With Prenatal Antiepileptic Drug (AED) Exposure in Monotherapy at High and Low Dose vs No Prenatal AED Exposure **eTable 5.** Risk of Intellectual Disability and Combined Outcome of Intellectual Disability and Delayed Childhood Milestones Associated With Prenatal Valproate Exposure Excluding Children With Congenital Malformations
