# Supplementary material for: Association of Prenatal Exposure to Valproate and Other Antiepileptic Drugs With Intellectual Disability and Delayed Childhood Milestones
Source: JAMA Netw Open. 2020 Nov 10;3(11):e2025570. doi: 10.1001/jamanetworkopen.2020.25570 (PMC7656282; doi:10.1001/jamanetworkopen.2020.25570)
Supplement: Supplement. — eAppendix. Underlying Disorders for Valproate Use During Pregnancy Without Maternal Epilepsy Diagnosis eTable 1. Risk of Intellectual Disability and Combined Outcome of Intellectual Disability and Delayed Childhood Milestones Associated With Prenatal Antiepileptic Drugs in Monotherapy Exposure vs Prenatal Lamotrigine in Monotherapy Exposure eTable 2. Risk of Intellectual Disability and Combined Outcome of Intellectual Disability and Delayed Childhood Milestones Associated With Valproate Use During Pregnancy vs Valproate Use Prior to Pregnancy eTable 3. Risk of Intellectual Disability and Combined Outcome of Intellectual Disability and Delayed Childhood Milestones Associated With Prenatal Antiepileptic Drug (AED) Exposure in Polytherapy and Monotherapy vs No Prenatal AED Exposure eTable 4. Risk of Intellectual Disability and Combined Outcome of Intellectual Disability and Delayed Childhood Milestones Associated With Prenatal Antiepileptic Drug (AED) Exposure in Monotherapy at High and Low Dose vs No Prenatal AED Exposure eTable 5. Risk of Intellectual Disability and Combined Outcome of Intellectual Disability and Delayed Childhood Milestones Associated With Prenatal Valproate Exposure Excluding Children With Congenital Malformations [file jamanetwopen-e2025570-s001.pdf]

Supplemental Online Content

Daugaard CA, Pedersen L, Sun Y, Dreier JW, Christensen J. Association of prenatal exposure to valproate and other antiepileptic drugs with intellectual disability and delayed childhood milestones. *JAMA Netw Open*. 2020;3(11):e2025570. doi:10.1001/jamanetworkopen.2020.25570

eAppendix. Underlying Disorders for Valproate Use During Pregnancy Without Maternal Epilepsy Diagnosis
eTable 1. Risk of Intellectual Disability and Combined Outcome of Intellectual Disability and Delayed Childhood Milestones Associated With Prenatal Antiepileptic Drugs in Monotherapy Exposure vs Prenatal Lamotrigine in Monotherapy Exposure
eTable 2. Risk of Intellectual Disability and Combined Outcome of Intellectual Disability and Delayed Childhood Milestones Associated With Valproate Use During Pregnancy vs Valproate Use Prior to Pregnancy
eTable 3. Risk of Intellectual Disability and Combined Outcome of Intellectual Disability and Delayed Childhood Milestones Associated With Prenatal Antiepileptic Drug (AED) Exposure in Polytherapy and Monotherapy vs No Prenatal AED Exposure
eTable 4. Risk of Intellectual Disability and Combined Outcome of Intellectual Disability and Delayed Childhood Milestones Associated With Prenatal Antiepileptic Drug (AED) Exposure in Monotherapy at High and Low Dose vs No Prenatal AED Exposure
eTable 5. Risk of Intellectual Disability and Combined Outcome of Intellectual Disability and Delayed Childhood Milestones Associated With Prenatal Valproate Exposure Excluding Children With Congenital Malformations

This supplemental material has been provided by the authors to give readers additional information about their work.

© 2020 Daugaard CA et al. *JAMA Network Open.*

Appendix. Underlying Disorders for Valproate Use During Pregnancy Without Maternal Epilepsy Diagnosis

We assessed the underlying disorders for use of valproate in the pregnancies where the mother did not have an epilepsy diagnosis, but was taking valproate in pregnancy. We included the following ICD10 diagnoses in our assessment:

G43 Migraine (n < 5);

F00-F09 Organic, including symptomatic, mental disorders (n = 0);

F10-F19 Mental and behavioural disorders due to psychoactive substance use (n = 5);

F20-F29 Schizophrenia, schizotypal and delusional disorders (n = 5);

F30-F39 Mood [affective] disorders (n = 13);

F40-F48 Neurotic, stress-related and somatoform disorders (n = 9);

F50-F59 Behavioural syndromes associated with physiological disturbances and physical factors (n < 5);

F60-F69 Disorders of adult personality and behavior (n = 5);

F70-F79 Mental retardation (n = 0);

F80-F89 Disorders of psychological development (n = 0);

F90-F98 Behavioural and emotional disorders with onset usually occurring in childhood and adolescence (n = 5);

F99-F99 Unspecified mental disorder (n = 5).

© 2020 Daugaard CA et al. *JAMA Network Open.*

eTable 1. Risk of Intellectual Disability and Combined Outcome of Intellectual Disability and Delayed Childhood Milestones Associated With Prenatal Antiepileptic Drugs in Monotherapy Exposure vs Prenatal Lamotrigine in Monotherapy Exposure

Hazard ratio of intellectual disability (ID)								
Monotherapy exposure to antiepileptic drugs	Live births (Number)	Person-years at risk	ID (Number)	Incidence (Cases per 1,000 person-years (95% CI)	Hazard ratio, crude (95% CI)		Hazard ratio, adjusted* (95% CI)	
Exposed to valproate	431	5,398	17	3.1 (2.0-5.1)	3.99	(1.85-8.62)	4.91	(2.09-11.55)
Exposed to carbamazepine	423	5,650	15	2.7 (1.6-4.4)	3.13	(1.48-6.60)	3.30	(1.51-7.24)
Exposed to clonazepam	314	3,744	6	1.6 (0.7-3.6)	2.03	(0.77-5.36)	1.90	(0.70-5.17)
Exposed to oxcarbazepine	372	4,656	12	2.6 (1.5-4.5)	2.97	(1.35-6.52)	3.53	(1.40-8.90)
Exposed to lamotrigine	1,383	12,244	10	0.8 (0.4-1.5)	1.00	(ref)	1.00	(ref)

*Adjusted for maternal age at conception, maternal psychiatric history, maternal epilepsy status, maternal diabetes status, sex of child, year of birth of child, and parity.

© 2020 Daugaard CA et al. *JAMA Network Open.*

eTable 1. (Continued)

Hazard ratio of the combined outcome of intellectual disability (ID) and delayed childhood milestones (CM)								
Monotherapy exposure to antiepileptic drugs	Live births (Number)	Person-years at risk	ID and CM (Number)	Incidence (Cases per 1,000 person-years (95% CI)	Hazard ratio, crude (95% CI)		Hazard ratio, adjusted* (95% CI)	
Exposed to valproate	431	5,184	38	7.3 (5.3-10.1)	3.86	(2.40-6.23)	4.58	(2.68-7.81)
Exposed to carbamazepine	423	5,582	18	3.2 (2.0-5.1)	1.69	(0.97-2.95)	1.77	(0.96-3.25)
Exposed to clonazepam	314	3,648	15	4.1 (2.5-6.8)	2.09	(1.14-3.84)	2.00	(1.06-3.77)
Exposed to oxcarbazepine	372	4,614	16	3.5 (2.1-5.7)	1.71	(0.96-3.05))	2.01	(1.01-4.00)
Exposed to lamotrigine	1,383	12,104	29	2.4 (1.7-3.4)	1.00	(ref)	1.00	(ref)

*Adjusted for maternal age at conception, maternal psychiatric history, maternal epilepsy status, maternal diabetes status, sex of child, year of birth of child, and parity.

© 2020 Daugaard CA et al. *JAMA Network Open.*

eTable 2. Risk of Intellectual Disability and Combined Outcome of Intellectual Disability and Delayed Childhood Milestones Associated With Valproate Use During Pregnancy vs Valproate Use Prior to Pregnancy

Hazard ratio of intellectual disability (ID)							
Valproate exposure	Live births (Number)	Person-years at risk	ID (number)	Hazard ratio, crude (95% CI)		Hazard ratio, adjusted* (95% CI)	
During pregnancy	580	7,153	23	1.82	(0.96-3.46)	1.94	(0.99-3.80)
Prior to pregnancy	719	9,051	16	1.00	Ref	1.00	Ref
Hazard ratio of the combined outcome of intellectual disability (ID) and delayed childhood milestones (CM)							
Valproate exposure	Live births (Number)	Person-years at risk	ID and CM (number)	Hazard ratio, crude (95% CI)		Hazard ratio, adjusted* (95% CI)	
During pregnancy	580	6,816	58	2.14	(1.41-3.26)	2.25	(1.46-3.47)
Prior to pregnancy	719	8,873	35	1.00	Ref	1.00	Ref

*Adjusted for maternal age at conception, maternal psychiatric history, maternal epilepsy status, maternal diabetes status, sex of child, year of birth of child, and parity.

© 2020 Daugaard CA et al. *JAMA Network Open.*

**eTable 3.** Risk of Intellectual Disability and Combined Outcome of Intellectual Disability and Delayed Childhood Milestones Associated With Prenatal Antiepileptic Drug (AED) Exposure in Polytherapy and Monotherapy vs No Prenatal AED Exposure							
Hazard ratio of intellectual disability (ID)							
AED	AED exposure	Live births (Number)	Incidence (Cases per 1,000 person-years) (95% CI)	Hazard ratio, crude (95% CI)		Hazard ratio, adjusted* (95% CI)	
Valproate	Monotherapy	431	3.1 (2.0-5.1)	4.65	(2.89-7.49)	4.42	(2.75-7.11)
	Polytherapy	149	3.4 (1.5-7.6)	5.12	(2.28-11.50)	4.99	(2.21-11.30)
	No exposure to AEDs	899,941	0.7 (0.6-0.7)	1.00	(ref)	1.00	(ref)
Carbamazepine	Monotherapy	423	2.7 (1.6-4.4)	3.88	(2.34-6.44)	3.84	(2.32-6.38)
	Polytherapy	81	1.8 (0.5-7.4)	2.73	(0.69-0.70)	2.60	(0.66-10.14)
	No exposure to AEDs	899,941	0.7 (0.6-0.7)	1.00	(ref)	1.00	(ref)
Clonazepam	Monotherapy	314	1.6 (0.7-3.6)	2.41	(1.09-5.34)	2.41	(1.09-5.35)
	Polytherapy	130	4.7 (2.2-9.8)	7.04	(3.35-14.80)	7.70	(3.68-16.08)
	No exposure to AEDs	899,941	0.7 (0.6-0.7)	1.00	(ref)	1.00	(ref)
Lamotrigine	Monotherapy	1,383	0.8 (0.4-1.5)	1.30	(0.70-2.42)	1.33	(0.71-2.48)
	Polytherapy	363	2.9 (1.6-5.3)	4.52	(2.50-8.16)	4.53	(2.52-8.15)
	No exposure to AEDs	899,941	0.7 (0.6-0.7)	1.00	(ref)	1.00	(ref)
Oxcarbazepine	Monotherapy	372	2.6 (1.5-4.5)	3.82	(2.17-6.72)	3.70	(2.11-6.51)

© 2020 Daugaard CA et al. *JAMA Network Open.*

	Polytherapy	120	2.8 (1.1-7.5)	4.21	(1.57-11.32)	3.95	(1.47-10.63)
	No exposure to AEDs	899,941	0.7 (0.6-0.7)	1.00	(ref)	1.00	(ref)

Person-years at risk and number of persons with ID and CM omitted because some cells contained < 5 persons.

*Adjusted for maternal age at conception, maternal psychiatric history, maternal epilepsy status, maternal diabetes status, sex of child, year of birth of child, and parity.

© 2020 Daugaard CA et al. *JAMA Network Open.*

eTable 3. (Continued)
Hazard ratio of the combined outcome of intellectual disability (ID) and delayed childhood milestones (CM)

AED	AED exposure	Live births (Number)	Incidence (Cases per 1,000 person-years) (95% CI)	Hazard ratio, crude (95% CI)		Hazard ratio, adjusted* (95% CI)	
Valproate	Monotherapy	431	7.3 (5.3-10.1)	5.35	(3.89-7.38)	5.28	(3.82-7.28)
	Polytherapy	149	12.3 (7.9-19.0)	8.68	(5.56-13.55)	8.95	(5.71-14.04)
	No exposure to AEDs	899,941	1.4 (1.4-1.5)	1.00	(ref)	1.00	(ref)
Carbamazepine	Monotherapy	423	3.2 (2.0-5.1)	2.42	(1.53-3.83)	2.49	(1.58-3.94)
	Polytherapy	81	5.7 (2.5-12.6)	4.25	(1.89-9.58)	4.26	(1.90-9.58)
	No exposure to AEDs	899,941	1.4 (1.4-1.5)	1.00	(ref)	1.00	(ref)
Clonazepam	Monotherapy	314	4.1 (2.5-6.8)	2.92	(1.76-4.84)	2.99	(1.80-4.96)
	Polytherapy	130	10.8 (6.5-18.0)	7.59	(4.54-12.67)	8.20	(4.88-13.76)
	No exposure to AEDs	899,941	1.4 (1.4-1.5)	1.00	(ref)	1.00	(ref)
Lamotrigine	Monotherapy	1,383	2.4 (1.7-3.4)	1.50	(1.04-2.16)	1.45	(1.01-2.09)
	Polytherapy	363	8.7 (6.1-12.3)	5.77	(4.06-8.21)	5.57	(3.87-8.02)
	No exposure to AEDs	899,941	1.4 (1.4-1.5)	1.00	(ref)	1.00	(ref)
Oxcarbazepine	Monotherapy	372	3.5 (2.1-5.7)	2.55	(1.57-4.13)	2.51	(1.54-4.10)

© 2020 Daugaard CA et al. *JAMA Network Open.*

	Polytherapy	120	8.2 (4.6-14.8)		5.84	(3.23-10.56)	5.27	(2.75-10.10)
	No exposure to AEDs	899,941	1.4 (1.4-1.5)		1.00	(ref)	1.00	(ref)

Person-years at risk and number of persons with ID and CM omitted because some cells contained < 5 persons.

*Adjusted for maternal age at conception, maternal psychiatric history, maternal epilepsy status, maternal diabetes status, sex of child, year of birth of child, and parity.

© 2020 Daugaard CA et al. *JAMA Network Open.*

eTable 4. Risk of Intellectual Disability and Combined Outcome of Intellectual Disability and Delayed Childhood Milestones Associated With Prenatal Antiepileptic Drug (AED) Exposure in Monotherapy at High and Low Dose vs No Prenatal AED Exposure

Hazard ratio of intellectual disability (ID)							
AED	AED exposure	Live births (Number)	Incidence (Cases per 1,000 person-years) (95% CI)	Hazard ratio, crude (95% CI)		Hazard ratio, adjusted* (95% CI)	
Valproate	High dose (>750 mg)	213	5.5 (3.3-9.1)	8.09	(4.88-13.43)	7.96	(4.80-13.19)
	Low dose (≤750 mg)	218	0.7 (0.2-3.0)	1.12	(0.28-4.45))	1.01	(0.25-4.08)
	No exposure	899,941	0.7 (0.6-0.7)	1.00	(ref)	1.00	(ref)
Carbamazepine	High dose (>500 mg)	259	2.3 (1.1-4.5)	3.32	(1.66-6.62)	3.50	(1.75-6.99))
	Low dose (≤500 mg)	164	3.3 (1.6-6.9)	4.88	(2.35-10.14)	4.38	(2.10-9.15)
	No exposure	899,941	0.7 (0.6-0.7)	1.00	(ref)	1.00	(ref)
Clonazepam	High dose (>4 mg)	31	NA	NA	NA	NA	NA
	Low dose (≤4 mg)	283	1.8 (0.8-4.0)	2.73	(1.23-6.07)	2.72	(1.22-6.06)
	No exposure	899,941	0.7 (0.6-0.7)	1.00	(ref)	1.00	(ref)
Lamotrigine	High dose (>150 mg)	911	0.6 (0.3-1.5)	0.98	(0.41-2.36)	1.04	(0.43-2.50)
	Low dose (≤150 mg)	472	1.2 (0.5-2.9)	1.94	(0.80-4.66)	1.85	(0.77-4.45)

© 2020 Daugaard CA et al. *JAMA Network Open.*

	No exposure	899,941	0.7 (0.6-0.7)	1.00	(ref)	1.00	(ref)
Oxcarbazepine	High dose (>1,000 mg)	212	2.7 (1.3-5.6)	4.01	(1.92-8.38)	4.14	(1.97-8.68)
	Low dose (≤1,000 mg)	160	2.4 (1.0-5.9)	3.61	(1.51-8.63)	3.25	(1.37-7.71)
	No exposure	899,941	0.7 (0.6-0.7)	1.00	(ref)	1.00	(ref)

Person-years at risk and number of persons with ID and CM omitted because some cells contained < 5 persons. NA (none exposed)

*Adjusted for maternal age at conception, maternal psychiatric history, maternal epilepsy status, maternal diabetes status, sex of child, year of birth of child, and parity.

© 2020 Daugaard CA et al. *JAMA Network Open.*

eTable 4. (Continued)

Hazard ratio of the combined outcome of intellectual disability (ID) and delayed childhood milestones (CM)

AED	AED exposure	Live births (Number)	Incidence (Cases per 1,000 person-years) (95% CI)	Hazard ratio, crude (95% CI)		Hazard ratio, adjusted* (95% CI)	
Valproate	High dose (>750 mg)	213	12.6 (8.9-17.8)	9.23	(6.49-13.11)	9.47	(6.64-13.49)
	Low dose (≤750 mg)	218	2.3 (1.0-5.1)	1.67	(0.75-3.70)	1.57	(0.70-3.50)
	No exposure	899,941	1.4 (1.4-1.5)	1.00	(ref)	1.00	(ref)
Carbamazepine	High dose (>500 mg)	259	2.9 (1.5-5.3)	2.17	(1.17-4.03)	2.34	(1.26-4.34)
	Low dose (≤500 mg)	164	3.8 (1.9-7.6)	2.81	(1.41-5.58)	2.68	(1.34-5.35)
	No exposure	899,941	1.4 (1.4-1.5)	1.00	(ref)	1.00	(ref)
Clonazepam	High dose (>4 mg)	31	4.8 (1.2-19.2)	3.65	(0.91-14.69)	4.05	(1.03-15.93)
	Low dose (≤4 mg)	283	4.0 (2.3-6.9)	2.83	(1.65-4.87)	2.88	(1.67-4.95)
	No exposure	899,941	1.4 (1.4-1.5)	1.00	(ref)	1.00	(ref)
Lamotrigine	High dose (>150 mg)	911	1.6 (0.9-2.8)	1.00	(0.58-1.73)	1.01	(0.59-1.74)
	Low dose (≤150 mg)	472	4.0 (2.4-6.5)	2.46	(1.51-4.01)	2.25	(1.38-3.66)
	No exposure	899,941	1.4 (1.4-1.5)	1.00	(ref)	1.00	(ref)
Oxcarbazepine	High dose (>1,000 mg)	212	3.9 (2.1-7.2)	2.79	(1.50-5.16)	2.89	(1.56-5.37)

© 2020 Daugaard CA et al. *JAMA Network Open.*

	Low dose (≤1,000 mg)	160	3.0 (1.3-6.6)		2.18	(0.98-4.85)	2.07	(0.94-4.57)
	No exposure	899,941	1.4 (1.4-1.5)		1.00	(ref)	1.00	(ref)

Person-years at risk and number of persons with ID and CM omitted because some cells contained < 5 persons.

*Adjusted for maternal age at conception, maternal psychiatric history, maternal epilepsy status, maternal diabetes status, sex of child, year of birth of child, and parity.

© 2020 Daugaard CA et al. *JAMA Network Open.*

eTable 5. Risk of Intellectual Disability and Combined Outcome of Intellectual Disability and Delayed Childhood Milestones Associated With Prenatal Valproate Exposure Excluding Children With Congenital Malformations

Hazard ratio of intellectual disability (ID)							
Valproate exposure	Live births (Number)	Person-years at risk	ID (Number)	Hazard ratio, crude (95% CI)		Hazard ratio, adjusted* (95% CI)	
Exposed to valproate	512	6,401	16	4.23	(2.59-6.92)	4.06	(2.48-6.63)
Not exposed to valproate	873,710	9,905,987	5,691	1.00	(ref)	1.00	(ref)
Hazard ratio of the combined outcome of intellectual disability (ID) and delayed childhood milestones (CM)							
Valproate exposure	Live births (Number)	Person-years at risk	ID and CM (Number)	Hazard ratio, crude (95% CI)		Hazard ratio, adjusted* (95% CI)	
Exposed to valproate	512	6,232	36	4.86	(3.50-6.75)	4.85	(3.49-6.74)
Not exposed to valproate	873,710	9,850,710	12,155	1.00	(ref)	1.00	(ref)

*Adjusted for maternal age at conception, maternal psychiatric history, maternal epilepsy status, maternal diabetes status, sex of child, year of birth of child, and parity.

© 2020 Daugaard CA et al. *JAMA Network Open.*
